# Platelet-Rich Plasma in Pediatric Surgery: A Comprehensive Review

**DOI:** 10.3390/children11080971

**Published:** 2024-08-12

**Authors:** Marco Di Mitri, Simone D’Antonio, Edoardo Collautti, Annalisa Di Carmine, Michele Libri, Tommaso Gargano, Mario Lima

**Affiliations:** Pediatric Surgery Department, IRCCS Azienda Ospedaliero-Universitaria di Bologna, Via Massarenti 11, 40138 Bologna, Italy; marcodimitri14@gmail.com (M.D.M.); simone.dantonio@aosp.bo.it (S.D.); edoardo.collautti@studio.unibo.it (E.C.); annalisa.dicarmine@studio.unibo.it (A.D.C.); michele.libri@aosp.bo.it (M.L.); tommaso.gargano2@unibo.it (T.G.)

**Keywords:** Platelet-Rich Plasma (PRP), pediatric surgery, hypospadias, pilonidal sinus, fistula, children

## Abstract

Platelet-Rich Plasma (PRP) therapy has become a promising treatment option in pediatric surgery, offering a novel approach to tissue repair and regeneration. Obtained from the patient’s own blood or umbilical cord blood (CB), PRP is a concentrated form of plasma enriched with platelets, growth factors, and cytokines essential for stimulating tissue healing. This systematic review explores the applications of PRP therapy in pediatric surgical procedures, focusing on its role in promoting wound healing, reducing postoperative complications, and enhancing patient outcomes. A systematic literature overview was conducted in accordance with PRISMA guidelines, encompassing studies published between 2004 and 2024. The research has identified different fields of application for PRP therapy in pediatric surgery, including treatment of pilonidal sinus and hypospadias repair. Key findings from clinical studies and randomized controlled trials are summarized, highlighting the efficacy of PRP therapy in accelerating wound healing, reducing pain, and improving patient recovery. Despite promising results, challenges and controversies surrounding PRP therapy persist, including variability in preparation protocols and optimal dosing regimens. The safety of PRP therapy in pediatric patients is also discussed, emphasizing its autologous nature and minimal risk of adverse reactions. In summary, this review highlights the role of PRP therapy as a safe and effective therapeutic approach in pediatric surgery, while further research to standardize protocols and elucidate optimal treatment strategies are still necessary.

## 1. Introduction

Platelet-Rich Plasma (PRP) therapy has become a promising treatment in the domain of regenerative medicine, offering a versatile approach to tissue repair and healing across various medical disciplines [1]. Extracted from the patient’s own blood (autologous) or from donated blood (heterologous), PRP is a concentrated form of plasma enriched with platelets, growth factors, cytokines, and other bioactive molecules essential to stimulate tissue regeneration, improving wound healing [2]. It delivers a high concentration of growth factors and cytokines directly to the site of injury, and by such means, accelerates the healing process, reducing inflammation, and enhancing tissue repair, thereby alleviating pain and restoring function. In fact, the regenerative properties of PRP stimulate collagen synthesis, increase dermal thickness, and improve vascularity, leading to smoother, firmer, and more youthful-looking skin. In recent years, PRP has witnessed a surge in interest and adoption owing to its favorable safety profile, minimally invasive nature, and promising clinical outcomes [3]. The autologous nature is pivotal in the production of PRP, as it eliminates the risk of immune rejection or transmission of infectious agents, and enhances its safety profile when compared to other allogeneic or xenogeneic therapies [4]. Furthermore, PRP offers a cost-effective and readily available treatment modality, leveraging the patient’s own biological resources to facilitate healing and tissue regeneration [5]. Moreover, the application of PRP extends beyond traditional medical specialties, finding utility in dental and maxillofacial surgery. In such fields, PRP has proved to be able to enhance bone regeneration, accelerate wound healing, and improve outcomes in implant dentistry and periodontal therapy [1]. Pediatric surgery often involves delicate procedures such as anatomical reconstruction of congenital malformations, and the healing process in pediatric patients can be challenging for clinicians. Despite the burgeoning interest and widespread adoption of PRP therapy, several challenges and controversies persist. Foremost among these is the lack of standardization in PRP preparation protocols, resulting in variability in the composition and bioactivity of PRP formulations across studies and clinical settings. Additionally, the optimal dosing regimens, frequency of administration, and timing of PRP injections remain topics of debate, necessitating further research to elucidate the most effective treatment strategies. In this paper, we present a review of PRP applications in pediatric surgery and introduce a new application of PRP in a pediatric patient.

## 2. Methods

**Figure 1:**
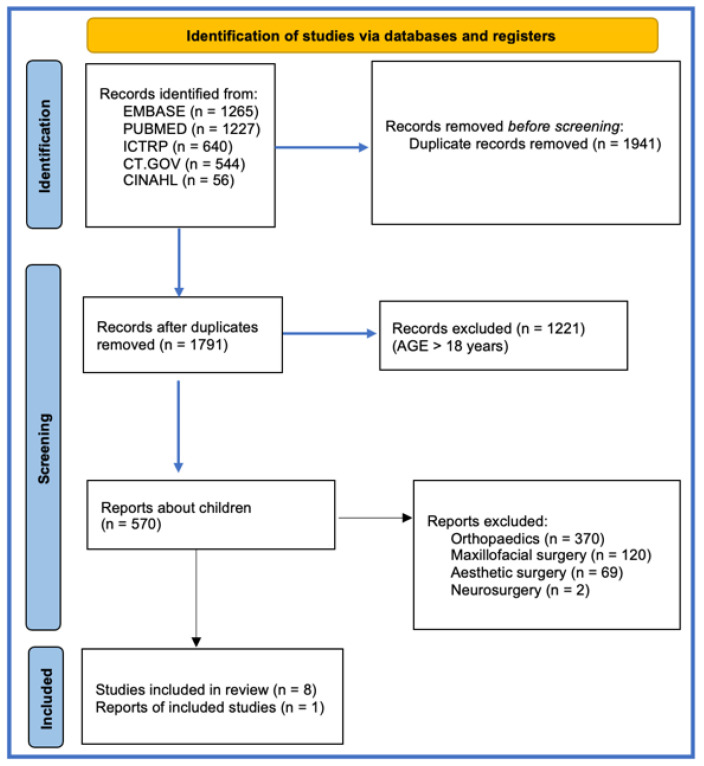
PRISMA statement.

We conducted a systematic literature review of PubMed, Embase, ICTRP, ClinicalTrials.gov, CINAHL, and Scopus in February 2024, following the Preferred Reporting Items for Systematic Reviews and Meta-Analysis (PRISMA) guidelines (Figure 1). The search was performed using the term “platelet-rich plasma” in all fields. We included papers published between January 2004 and January 2024. The research criteria were limited to children, English language, and studies involving human subjects. All titles and abstracts retrieved from the initial search were independently evaluated for relevance by two reviewers. Studies deemed relevant underwent full-text review. All stages of the review process, including screening, selection, and data extraction, were conducted independently by two reviewers. Any discrepancies were resolved through discussion and consensus.

## 3. Platelet-Rich Plasma Preparation

PRP can be obtained from autologous or heterologous blood or sampled from umbilical CB. The first step consists of an informational interview with the patient and/or the parents about PRP application, its benefits, and potential risks. Based on patient characteristics, it’s possible to choose either autologous or umbilical CB-derived PRP. PRP from autologous blood is preferred for patients over 12 months of age undergoing surgical procedures under general anesthesia.

For other conditions, such as postoperative medications or patients under 12 months of age, the use of PRP derived from umbilical CB is preferred.

PRP processing consists of the following steps:

1. Obtaining informed consent from the patient/parents.

2. Blood collection:

Selecting a suitable vein for venipuncture.

Cleaning the area with an antiseptic solution to reduce the risk of infection.

Using a sterile needle and collection tube to draw a specific volume of blood, typically between 10 to 20 mL, depending on the intended application and the patient’s needs.

3. Blood processing:

Transferring the collected blood into a sterile, closed-system PRP preparation kit or centrifuge tubes designed for PRP isolation.

Centrifuging the blood at a specific speed and duration to separate its components based on their densities.

The centrifugation process typically separates whole blood into three layers: red blood cells (bottom layer), PRP (middle layer), and platelet-poor plasma (PPP) or plasma (top layer).

4. PRP extraction:

After centrifugation, the PRP layer is carefully extracted using a sterile syringe or pipette. The PRP layer is located just above the layer of red blood cells.

It is important to avoid any contact with the interface between the PRP and the underlying red blood cell layer to prevent contamination with red blood cells.

5. PRP activation:

Some protocols involve the activation of PRP using additives like calcium chloride or thrombin to release growth factors and enhance its regenerative properties.

Activation can occur either before or after PRP application, depending on the specific treatment protocol.

6. Applications:

Administering the prepared PRP using appropriate techniques based on the intended treatment area.

PRP can be injected directly into target tissues, applied topically, or used in conjunction with other medical procedures like microneedling or surgical procedures.

7. Post-treatment care:

Providing the patient with post-treatment instructions, including information about potential side effects and activities to avoid.

Scheduling follow-up appointments to monitor the patient’s progress and assess the effectiveness of PRP therapy.

By following these steps, healthcare providers can prepare and administer autologous PRP effectively, leveraging its regenerative properties for various therapeutic applications. It’s important to note that specific protocols may vary depending on factors such as the PRP preparation system used and the intended treatment goals.

PRP from CB is prepared with the CB units collected, after the mothers’ informed consent, into plastic bags containing 25 mL of citrate-phosphate-dextrose anticoagulant by trained midwives, before and after placental delivery. The units are processed within 48 h from collection. After two centrifugation steps and the removal of the supernatant PPP, the platelet concentrate in PRP ranges between 800 × 10^9^/L and 1200 × 10^9^/L. The PRP units are then transferred to a storage bag and cryopreserved in a mechanical freezer at a temperature below −40 °C. Microbiological control is always performed on the PPP. To obtain a CB platelet the PRP thaws at 37 °C in a water-bath and is activated with calcium gluconate with or without thrombin in a 37 °C incubator. The Platelet-Rich Plasma gel (PRP-G) is then sent to the pediatric surgery department for application.

## 4. Applications

### 4.1. Pilonidal Sinus

Literature reports two papers regarding the application of PRP-G as an adjuvant to improve wound healing after pilonidal sinus surgery.

Mohamadi et al. enrolled 110 patients divided into two groups: a study group (n = 55) treated with PRP-G and the control group. All the patients were subject to an open surgical approach involving an elliptical incision performed by an experienced surgeon. Upon completion of the procedure, autologous PRP-G was applied to patients belonging to the study group while those in the control group were managed with standard medication, including absorbent sterile cotton gauze. They chose 9 weeks as the endpoint of the study and showed a statistically significant improvement in patients treated with PRP-G, including healing time (*p* < 0.001), pain duration (*p* < 0.001), and antibiotic consumption (*p* < 0.001). Moreover, 3–5 days before healing they performed a biopsy of the tissue to analyze the process of angiogenesis, and it showed an improvement in the PRP-G group (*p* < 0.001) [6]. A randomized controlled clinical trial by Boztug et al. showed interesting results applying PRP to heal pilonidal sinus. They collected 49 patients who were divided into three groups: group A (n = 18) as the control group, including patients treated by open technique; group B (n = 22), including patients who underwent open surgery followed by the application of PRP; and group C (n = 9), including patients who underwent minimally invasive curettage of the sinus tract and PRP application. To standardize patients across different surgical procedures, recovery time based on the cavity extension (day/cc) was used as a parameter for assessing pilonidal sinus severity. The cavity volume of the sinus was comparable among groups, but the recovery time per unit of cavity volume was significantly shorter in group B compared to group A (*p* < 0.001). Additionally, the need for painkillers was lower in groups B and C compared to group A (*p* < 0.001), and both PRP groups B and C experienced a significantly shorter time to return to normal activities compared to group A (*p* = 0.003 and *p* < 0.001, respectively). The authors concluded that surgeons should consider PRP application for pilonidal sinus treatment, as it improves postoperative recovery by accelerating the return to daily activities, reducing pain, and enhancing overall quality of life [7].

### 4.2. Hypospadias Repair

We found four papers reporting the use of PRP for hypospadias repair. Eryilmaz et al. collected 40 patients in a prospective study analyzing the role of PRP to reduce postoperative complications in children who underwent hypospadias repair. All patients underwent a Snodgrass urethroplasty for mid penile hypospadias, and the authors divided the sample into two groups: Group A (n = 20), who received the PRP application between the dartos flap and the skin; and Group B (n = 20), who underwent the standard procedure without the PRP application. They analyzed two follow-up endpoints: 1 month postoperatively, showing a rate of fistula for Group A and Group B, respectively, of 10% and 25% and a rate of stenosis, respectively, of 5% and 20%. The authors also found a significantly lower rate of infection in patients who were administered PRP (*p* < 0.05) 5 months postoperatively, showing no change in urethral fistula, stenosis, and infection rates in the group using PRP but an increase in urethral stenosis that was observed in the group without PRP. Based on these results, the authors concluded that PRP has the potential to prevent early and long-term postoperative complications occurring after hypospadias repair [8]. Another paper by Guinot et al. analyzed 33 patients who underwent the Tiersch–Duplay procedure for hypospadias repair. They applied autologous PRP to cover the urethroplasty, suturing the patch with polyglyconate 7/0. The authors followed the patients for 8 months, comparing the complications rate with a control group with the same characteristics. From their results, they found a lower rate of urethral fistula in the study group, but the differences were not statistically significant. With the limitation of the sample size, the authors concluded that PRP could be a useful way to cover the urethroplasty, especially in re-do cases without suitable foreskin, in circumcised hypospadias, in crippled cases, and in cases without healthy dartos tissue or when tunica vaginalis is no longer available owing to a previous testicular operation [9].

Similar results were reported by Mahmoud et al. that compared 90 patients treated for hypospadias plus application of PRP and 90 patients treated for hypospadias without the application of PRP.

At the end of the follow-up (6 months), they detected a lower rate of complications (urethrocutaneous fistula, partial superficial wound infection, partial glans dehiscence, meatal stenosis, and urethral stricture) in the PRP group (*p* < 0.05) [10].

### 4.3. Spontaneous Pneumothorax

Kimura et al., in a retrospective study, investigated the role of PRP from autologous blood in preventing postoperative recurrences in patients who underwent video-assisted thoracic surgery for spontaneous pneumothorax. They compared two groups: A (control) and B (PRP). PRP was applied to cover the staple lines. No report of operative morbidity or mortality, such as allergic reactions, was found. Comparing the two groups, they showed that the recurrence rate was significantly lower in Group B (*p* = 0.02), and the recurrence rate in patients younger than 25 years in Group A and Group B was significantly different (26.1 and 0.0%, respectively; *p* = 0.03) [11].

### 4.4. Chylothorax

Tashnizi et al. presented an innovative use of Platelet-Rich Plasma fibrin glue (PRP-FG) to treat the chylothorax in children following cavopulmonary connections. They injected PRP-FG into the pleural space through the chest tube, defining success as a volume of effusion below 50 mL/day after 2 days. The PRP-FG injection was repeated with the same volume if the effusion continued after 1 week. They achieved an incredible result, reaching a rate of successful treatment of 92%. Based on these results, although the sample was small, the authors concluded that treating the chylothorax with PRP-FG is safe and feasible [12].

### 4.5. Enterocutaneous Fistula

We documented the first use of PRP in an infant who developed an esophago-cutaneous fistula following esophagocoloplasty for esophageal atresia type A (Gross classification). Due to the significant gap between the upper and lower esophageal pouches (spanning more than three vertebral bodies), the patient underwent extra-thoracic, thoracoscopic-assisted elongation of the esophageal stumps using Foker’s technique. After 10 days, the anastomosis failed, resulting in a left cervicostomy. At 7 months of age, the patient underwent a procedure of esophageal replacement with colonic segment. On the 14th postoperative day, an esophago-cutaneous fistula developed above the proximal esophagus-colon anastomosis, causing excessive saliva leakage. Despite 2 months of advanced medication treatment, there was no improvement. Given the patient’s young age, we opted to use PRP-G derived from umbilical cord blood, prepared at the Emilia Romagna Cord Blood Bank in S. Orsola Hospital (Bologna, Italy). PRP-G was applied four times, with renewals every fourth day. This treatment led to complete closure of the fistula after the third application and also improved the quality of the scar [13].

### 4.6. Other Clinical Applications

Musculoskeletal Injuries: PRP injections have been explored as a treatment option for musculoskeletal injuries in children, including juvenile idiopathic arthritis, osteochondral defects, ligament injuries, and tendonitis. The growth factors and cytokines present in PRP are thought to promote tissue repair and regeneration, potentially aiding in the healing process of these injuries [14,15,16,17,18].

Congenital Anomalies: PRP may be utilized in the management of congenital anomalies, including conditions like spina bifida and congenital heart defects. While more research is needed in this area, PRP offers a potential adjunctive therapy to traditional management approaches in these complex conditions [19].

Wound Healing: PRP has been investigated for its role in promoting wound healing in pediatric patients, particularly in cases of chronic wounds or non-healing ulcers. The growth factors and stem cells present in PRP may accelerate the wound-healing process by promoting angiogenesis, collagen synthesis, and tissue regeneration [20,21,22,23].

Orthodontics and Dentistry: PRP has been explored in pediatric orthodontics and dentistry for its potential role in accelerating tooth movement, enhancing bone regeneration after tooth extraction, and promoting periodontal tissue regeneration. The growth factors and stem cells present in CBPRP may improve the outcomes of these dental procedures [24,25,26].

## 5. Safety

PRP therapy is widely recognized for its favorable safety profile, making it an attractive option for clinicians and patients alike. One of the primary advantages of PRP therapy is its autologous nature, whereby the patient’s own blood is used to prepare the compound. This feature eliminates the risk of immune rejection or allergic reactions, which are common concerns associated with allogeneic or xenogeneic therapies. Moreover, the preparation process of PRP involves centrifugation of the patient’s blood to concentrate platelets and growth factors, followed by the extraction of the PRP component. This extraction process typically excludes red blood cells, white blood cells, and other cellular components, focusing primarily on isolating platelets and their associated bioactive molecules. As a result, PRP formulations are relatively free from cellular contaminants, reducing the risk of immunogenic reactions and infectious transmission. In addition to its autologous nature and minimal risk of immunogenicity, PRP therapy is associated with a low rate of adverse events and complications. Clinical studies and systematic reviews evaluating the safety of PRP across various medical specialties have consistently reported a low incidence of adverse reactions, such as pain at the injection site, transient swelling, bruising, and erythema. These minor adverse events are typically self-limiting and resolve spontaneously within a few days following PRP administration. Furthermore, PRP therapy is minimally invasive and can be performed in an outpatient setting, further mitigating the risks associated with more invasive surgical procedures. The use of ultrasound or fluoroscopy guidance during PRP injections allows for precise targeting of the affected tissue, minimizing the risk of inadvertent injury to surrounding structures and enhancing the safety and efficacy of the procedure [12]. However, certain precautions should be taken when considering PRP therapy, particularly in patients with underlying medical conditions or contraindications. Patients with bleeding disorders, thrombocytopenia, or platelet dysfunction may not be suitable candidates for PRP therapy, as it can exacerbate bleeding tendencies and compromise hemostasis. Similarly, individuals with active infections, systemic illnesses, or malignancies may require careful evaluation and risk assessment before undergoing PRP treatment. Additionally, while PRP therapy is generally considered safe, its long-term effects and potential risks warrant further investigation. Longitudinal studies tracking patients’ outcomes and monitoring for any delayed adverse events or complications are essential for ensuring the continued safety and efficacy of PRP therapy. In conclusion, PRP therapy offers a safe and minimally invasive approach to tissue repair and regeneration across various medical specialties. Its autologous nature, minimal risk of immunogenicity, and low incidence of adverse events make PRP an attractive option for patients seeking natural and biocompatible treatments. However, careful patient selection, adherence to standardized protocols, and ongoing surveillance are imperative to mitigate potential risks and ensure the safe and effective use of PRP therapy in clinical practice [27,28].

## 6. Discussion

This review highlights the applications of PRP therapy in pediatric surgery, emphasizing its potential benefits across various surgical procedures. The findings from the studies analyzed indicate that PRP not only accelerates wound healing but also significantly reduces postoperative complications.

The evidence suggests that the biological properties of PRP—such as its rich content of growth factors—play a crucial role in enhancing tissue regeneration and healing [1]. In fact, Mahmoud et al. showed promising results in terms of reduction of complications rates in hypospadias repair, such as urethrocutaneous fistula, infection, partial glans dehiscence, meatal stenosis, and urethral stricture in patients treated by PRP compared with the control group [10]. According to these results, the study by Guinot showed a lower rate of uretheral fistula in patients who received PRP applications, emphasizing the role of PRP especially in patients who do not have enough skin for the urethra reconstruction [9]. Promising results have also been shown by Eryilmaz et al. that proved a significant lower rate of infection in patients who were administered PRP (*p* < 0.05) associated with a decrease of urethral stenosis [8]. For instance, in the management of pilonidal sinus, Boztug et al. showed an important role of PRP in reducing the recovery time, the postoperative painkillers needed, and the time to return to daily activities [7].

According to the results from Botzug et al., Mohamadi et al. showed a statistically significant improvement in patients with pilonidal sinus treated by PRP-G, including healing time, pain, and antibiotic consumption. They also showed an improvement in the angiogenesis on biopsy samples in the PRP-G group [6]. The results show promising outcomes, particularly in conditions like pilonidal sinus and hypospadias repair, where PRP applications have led to faster recovery times and improved patient satisfaction [6,7,8,9,10]. However, while the positive results are compelling, it is essential to consider the variability in PRP preparation protocols, which can affect the consistency and effectiveness of treatment across different clinical settings. Additionally, although the safety profile of PRP is favorable due to its autologous nature, careful patient selection remains paramount [29].

The absolute contraindications to the use of autologous PRP are related to blood disorders (platelet dysfunction syndrome, critical thrombocytopenia) and sepsis. In these cases, it is, however, possible to use PRP derived from heterologous blood [30].

This study would help validate the efficacy of PRP therapy across a broader population and establish definitive guidelines for its use in pediatric patients.

## 7. Conclusions

In conclusion, PRP therapy seems to represent a promising adjunctive treatment in pediatric surgery, offering a natural and biocompatible option for enhancing surgical outcomes. The clinical applications of PRP in pediatric surgery are diverse and promising. However, ongoing research is crucial to address existing challenges, standardize practices, and fully elucidate the optimal conditions under which PRP can be most effective. Careful patient selection, adherence to standardized protocols, and ongoing surveillance are imperative to mitigate potential risks and ensure the safe and effective use of PRP therapy in clinical practice.

Moreover, while the reviewed studies provide strong evidence for the effectiveness of PRP in specific pediatric surgical applications, there is a need for larger, multicentric clinical trials.

## Data Availability

Not applicable.

## Abbreviations

Platelet-Rich Plasma (PRP); Platelet-Rich Plasma Gel (PRP-G), Cord Blood (CB), Platelet-Poor Plasma (PPP), Platelet-Rich Plasma Fibrin Glue (PRP-FG), Cord Blood Platelet-Rich Plasma (CBPRP).

## References

[1] Everts P., Onishi K., Jayaram P., Lana J.F., Mautner K. (2020). Platelet-Rich Plasma: New Performance Understandings and Therapeutic Considerations in 2020. Int. J. Mol. Sci..

[2] Dohan Ehrenfest D.M., Rasmusson L., Albrektsson T. (2009). Classification of platelet concentrates: From pure platelet-rich plasma (P-PRP) to leucocyte- and platelet-rich fibrin (L-PRF). Trends Biotechnol..

[3] Bolton L. (2021). Platelet-Rich Plasma: Optimal Use in Surgical Wounds. Wounds Compend. Clin. Res. Pract..

[4] Tang J.Z., Nie M.J., Zhao J.Z., Zhang G.C., Zhang Q., Wang B. (2020). Platelet-rich plasma versus hyaluronic acid in the treatment of knee osteoarthritis: A meta-analysis. J. Orthop. Surg..

[5] Cartron A.M., Shaikh G. (2021). Platelet-Rich Plasma: Cure-All at What Cost?. Dermatol. Surg..

[6] Mohamadi S., Norooznezhad A.H., Mostafaei S., Nikbakht M., Nassiri S., Moghaddam K.A., Ghavamzadeh A., Kazemnejad A. (2019). A randomized controlled trial of effectiveness of platelet-rich plasma gel and regular dressing on wound healing time in pilonidal sinus surgery: Role of different affecting factors. Biomed. J..

[7] Boztug C.Y., Karaagac Akyol T., Benlice C., Koc M.A., Doganay Erdogan B., Ozcebe O.I., Kuzu M.A., Akyol C. (2021). Platelet-rich plasma treatment improves postoperative recovery in patients with pilonidal sinus disease: A randomized controlled clinical trial. BMC Surg..

[8] Eryilmaz R., Şimşek M., Aslan R., Beger B., Ertaş K., Taken K. (2020). The effect of plasma rich platelet graft on post-operative complications in mid-penile hypospadias. Andrologia.

[9] Guinot A., Arnaud A., Azzis O., Habonimana E., Jasienski S., Frémond B. (2014). Preliminary experience with the use of an autologous platelet-rich fibrin membrane for urethroplasty coverage in distal hypospadias surgery. J. Pediatr. Urol..

[10] Mahmoud A.Y., Gouda S., Gamaan I., Baky Fahmy M.A. (2019). Autologous platelet-rich plasma covering urethroplasty versus dartos flap in distal hypospadias repair: A prospective randomized study. Int. J. Urol. Off. J. Jpn. Urol. Assoc..

[11] Kimura M., Miyajima K., Kono T., Hayashi A., Iwaya K., Ikeda N. (2017). Effectiveness of Polyglycolic Acid Sheet Covering and Platelet-Rich Plasma after Video-Assisted Thoracic Surgery for Spontaneous Pneumothorax. Thorac. Cardiovasc. Surg..

[12] Alamdari D.H., Asadi M., Rahim A.N., Maddah G., Azizi S., Shahidsales S., Mehrabibahar M. (2018). Efficacy and Safety of Pleurodesis Using Platelet-Rich Plasma and Fibrin Glue in Management of Postoperative Chylothorax after Esophagectomy. World J. Surg..

[13] Di Mitri M., Chiastra G., Collautti E., D’Antonio S., Buzzi M., Bisanti C., Di Carmine A., Catania V., Libri M., Gargano T. (2024). Platelet-rich plasma therapy for postoperative esophageal fistula in a pediatric patient. J. Surg. Case Rep..

[14] Sampson S., Reed M., Silvers H., Meng M., Mandelbaum B. (2010). Injection of platelet-rich plasma in patients with primary and secondary knee osteoarthritis: A pilot study. Am. J. Phys. Med. Rehabil..

[15] Karjalainen T.V., Silagy M., O’Bryan E., Johnston R.V., Cyril S., Buchbinder R. (2021). Autologous blood and platelet-rich plasma injection therapy for lateral elbow pain. Cochrane Database Syst. Rev..

[16] Kaminski R., Kulinski K., Kozar-Kaminska K., Wielgus M., Langner M., Wasko M.K., Kowalczewski J., Pomianowski S. (2018). A Prospective, Randomized, Double-Blind, Parallel-Group, Placebo-Controlled Study Evaluating Meniscal Healing, Clinical Outcomes, and Safety in Patients Undergoing Meniscal Repair of Unstable, Complete Vertical Meniscal Tears (Bucket Handle) Augmented with Platelet-Rich Plasma. BioMed Res. Int..

[17] Lazzaretti Fernandes T., Taraballi F., Shao Z., Roessler P.P., Cardona-Ramírez S. Nonoperative and Operative Soft Tissue, Cartilage, and Bony Regeneration and Orthopaedic Biologics of the Elbow and Upper Extremity: An Orthoregeneration Network (ON) Foundation Review Authors. Arthrosc. J. Arthrosc. Relat. Surg..

[18] Paget L.D., Reurink G., de Vos R.J., Weir A., Moen M.H., Bierma-Zeinstra S.M., Stufkens S.A., Goedegebuure S., Krips R., Maas M. (2023). Platelet-Rich Plasma Injections for the Treatment of Ankle Osteoarthritis. Am. J. Sports Med..

[19] Joshi A.D., More S.N., Mhambre A.S. (2020). Treatment of a Chronic, Nonhealing Neuropathic Ulcer in a Pediatric Patient with Spinal Dysraphism: A Case Report. Wound Manag. Prev..

[20] Barwijuk M., Pankiewicz K., Gałaś A., Nowakowski F., Gumuła P., Jakimiuk A.J., Issat T. (2024). The Impact of Platelet-Rich Plasma Application during Cesarean Section on Wound Healing and Postoperative Pain: A Single-Blind Placebo-Controlled Intervention Study. Med. Kaunas. Lith..

[21] Napit I.B., Shrestha D., Choudhury S., Gkini E., Ilozumba O., Gill P., Bishop J., Neupane K., Adhikari A., Sartori J. (2024). A randomised Trial of Autologous Blood products, leukocyte and platelet-rich fibrin (L-PRF), to promote ulcer healing in LEprosy: The TABLE trial. PLoS Negl. Trop. Dis..

[22] Jin F., Li X., Chen J., Liu J., Wang Y. (2024). Clinical study on the role of platelet-rich plasma in human acellular dermal matrix with razor autologous skin graft repair of giant congenital pigmented nevus in children. J. Plast. Reconstr. Aesthetic Surg. JPRAS.

[23] Mohseni R., Sharif P.M., Khosravi A., Taheri A.R., Behfar M., Zarrabi M., Jafari L., Jafari F., Nikfetrat Z., Naji P. (2024). The Application of Umbilical Cord Blood-derived Platelet Gel for Skin Ulcers Associated with Chronic Graft-Versus-Host Disease in Pediatrics: A Randomized Trial. Transplant. Cell. Ther..

[24] Gupta S., Bhambri E., Sharma M., Shaikh M.A., Zope A., Thoke B., Sorokhaibam M. (2023). Does leukocyte-platelet-rich fibrin (L-PRF) cause long term acceleration in the rate of canine retraction? A split-mouth, two-arm parallel group, randomized control trial. Dent. Press. J. Orthod..

[25] Ammar A.M., Al-Sabbagh R., Hajeer M.Y. (2024). Evaluation of the effectiveness of the platelet-rich plasma compared to the injectable platelet-rich fibrin on the rate of maxillary canine retraction: A three-arm randomized controlled trial. Eur. J. Orthod..

[26] Derwich M., Mitus-Kenig M., Pawlowska E. (2021). Mechanisms of Action and Efficacy of Hyaluronic Acid, Corticosteroids and Platelet-Rich Plasma in the Treatment of Temporomandibular Joint Osteoarthritis—A Systematic Review. Int. J. Mol. Sci..

[27] Prokurat M., Grudnik K., Niemczyk W., Niemczyk S., Migas M., Wągrowska K., Lau K., Kasperczyk J. (2024). Platelet-Rich Plasma—A remedy present in every human being. History, functioning, and the benefits of therapy using it. Pol. Merkur. Lek. Organ. Pol. Tow. Lek..

[28] Toora E., Kulkarni R.G., Manivannan P., Sastry A.S., Basavarajegowda A., Sahoo D. (2023). Quality assessment of platelet concentrates prepared by platelet-rich plasma, buffy-coat, and apheresis methods in a tertiary care hospital in South India: A cross-sectional study. Asian J. Transfus. Sci..

[29] Paganelli A., Contu L., Condorelli A., Ficarelli E., Motolese A., Paganelli R., Motolese A. (2023). Platelet-Rich Plasma (PRP) and Adipose-Derived Stem Cell (ADSC) Therapy in the Treatment of Genital Lichen Sclerosus: A Comprehensive Review. Int. J. Mol. Sci..

[30] Jain N.K., Gulati M. (2016). Platelet-rich plasma: A healing virtuoso. Blood Res..

